# Deletion of African swine fever virus interferon inhibitors from the genome of a virulent isolate reduces virulence in domestic pigs and induces a protective response

**DOI:** 10.1016/j.vaccine.2016.08.011

**Published:** 2016-09-07

**Authors:** Ana Luisa Reis, Charles C. Abrams, Lynnette C. Goatley, Chris Netherton, Dave G. Chapman, Pedro Sanchez-Cordon, Linda K. Dixon

**Affiliations:** The Pirbright Institute, Ash Road, Pirbright, Woking, Surrey GU24 0NF, UK

**Keywords:** ASFV, Attenuation, MGF360, MGF530/505, Interferon

## Abstract

African swine fever virus (ASFV) encodes multiple copies of MGF360 and MGF530/505 gene families. These genes have been implicated in the modulation of the type I interferon (IFN) response. We investigated the effect of modulating the IFN response on virus attenuation and induction of protective immunity by deleting genes MGF360 (MGF360-10L, 11L, 12L, 13L, 14L) and MGF530/505 (MGF530/505-1R, 2R and 3R) and interrupting genes (MGF360-9L and MGF530/505-4R) in the genome of the virulent ASFV isolate Benin 97/1. Replication of this deletion mutant, BeninΔMGF, in porcine macrophages *in vitro* was similar to that of the parental virulent virus Benin 97/1 and the natural attenuated isolate OURT88/3, which has a similar deletion of MGF360 and 530/505 genes. Levels of IFN-β mRNA in macrophages infected with virulent Benin 97/1 isolate were barely detectable but high levels were detected in macrophages infected with OURT88/3 and intermediate levels in macrophages infected with BeninΔMGF. The data confirms that these MGF360 and MGF530/505 genes have roles in suppressing induction of type I IFN. Immunisation and boost of pigs with BeninΔMGF showed that the virus was attenuated and all pigs (5/5) were protected against challenge with a lethal dose of virulent Benin 97/1. A short transient fever was observed at day 5 or 6 post-immunisation but no other clinical signs. Following immunisation and boost with the OURT88/3 isolate 3 of 4 pigs were protected against challenge. Differences were observed in the cellular and antibody responses in pigs immunised with BeninΔMGF compared to OURT88/3. Deletion of IFN modulators is a promising route for construction of rationally attenuated ASFV candidate vaccine strains.

## 1 Introduction

African swine fever (ASF) is an acute haemorrhagic disease of domestic pigs that can have a devastating socio-economic impact in affected regions. Currently these include sub-Saharan Africa, Trans-Caucasus, Russian Federation, parts of E. Europe and Sardinia [1]. No vaccine is available limiting options for control [2], [3]. ASF is caused by a large, cytoplasmic, double-stranded DNA virus, African swine fever virus (ASFV) [4] with a genome varying between 170 and 193 kbp, mainly due to variation in the copy numbers of different multigene families (MGFs) [5].

Immunisation of pigs with an attenuated ASFV, OURT88/3, can protect against lethal challenge with isolates from the same genotype I and other genotypes [6], [7] and the protection is dependent on CD8^+^ cells [8]. This isolate has been used as a model to understand the correlates for induction of protection and was therefore included in this study for comparison. The OURT88/3 strain has a large contiguous deletion of genes MGF360-10L, 11L, 12L, 13L, 14L, MGF530/505-1R, 2R and truncation of gene MGF530/505-3R [9]. Deletion of similar MGF genes from the Pr4 genotype XX strain increased induction of type I interferon (IFN) and interferon stimulated genes in macrophages infected *in vitro*. It was suggested that this inability to control the IFN response was responsible for the attenuation of the deletion mutant in pigs [10]. In addition, deletion of these genes from a genotype II Georgia strain reduced virulence in pigs and induced protection against challenge [11]. In this study we investigated the impact of deleting or interrupting additional copies of MGF360 and 530/505 genes compared to those studied previously from a virulent genotype I isolate, Benin 97/1. MGF360-10L, 11L, 12L, 13L, 14L and MGF530/505-1R, 2R and 3R were deleted and MGF360-9L and MGF530/505-4R inactivated. This deletion mutant, BeninΔMGF was compared with the attenuated OURT88/3 strain, a well characterised model for induction of protection, by *in vitro* infections and immunisation and challenge in pigs. The results highlight differences between interferon induction and *in vivo* host responses.

## 2 Materials and methods

### 2.1 Viruses and cells

The Benin 97/1 and OURT88/3 isolates were described previously [6], [7], [9]. Viruses were cultured in porcine bone marrow (PBMs) or alveolar macrophages (PAMs). Virus titres were determined by haemadsorbtion (HAD_50_/ml) [12] or by immunofluorescence using antibodies against ASFV early protein p30 [13].

### 2.2 Construction of ASFV BeninΔMGF virus

**Supplementary Fig. 1:**
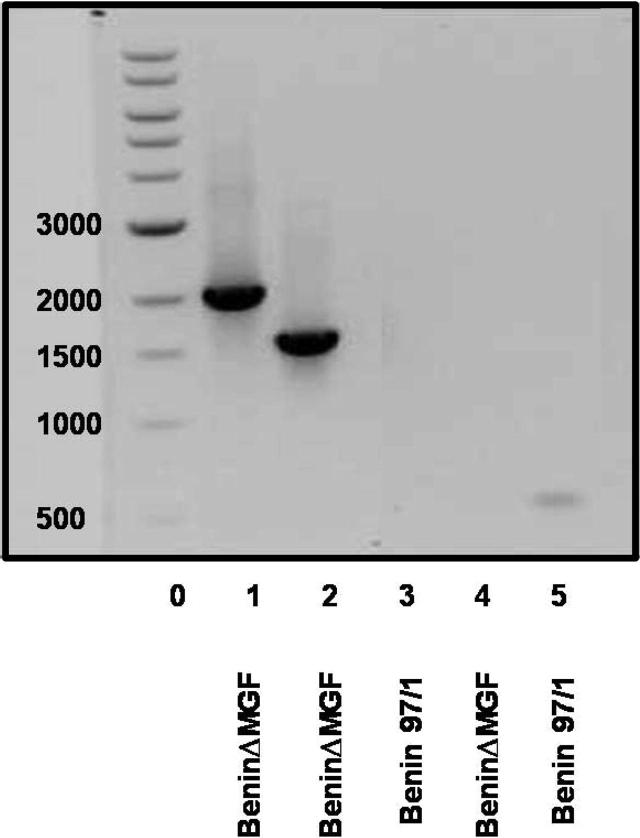


**Fig. 1:**
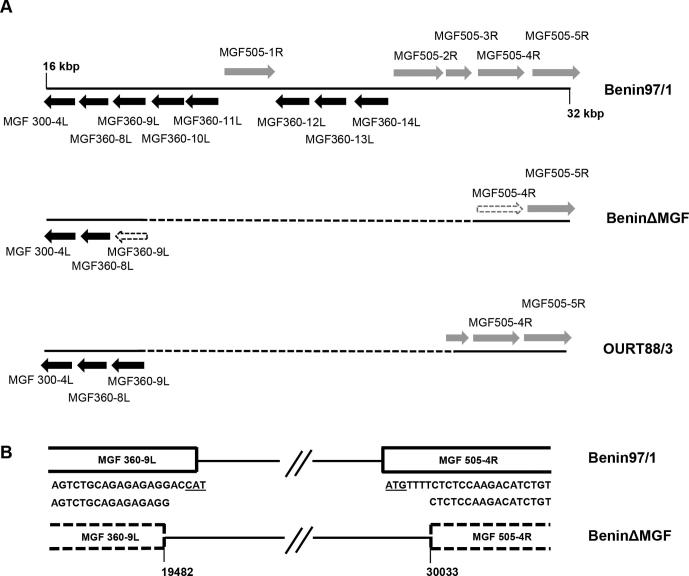
ASFV genome showing position of deletions in BeninΔMGF genome. Panel A shows a diagram of the left end of the genome of ASFV Benin 97/1 isolate between position 16 and 32 kbp. Open reading frames are indicated with arrows and labelled above or below. Below the position of deletions in BeninΔMGF and OURT88/3 strains are shown as a dashed line. The ORFs MGF360-9L and MGF530/505-4R which are interrupted in the BeninΔMGF isolate are outlined with dashed lines. Panel B shows the sequence of the Benin 97/1 isolate at the N-terminus of the MGF360-9L and MGF 530/505-4R genes in the upper panel and below the same region of the BeninΔMGF strain indicating the sequences deleted from the start of these ORFs.

Right and left genome fragments flanking genes MGF360-10L, 11L, 12L, 13L, 14L and MGF530/505-1R, 2R and 3R (Fig. 1) were amplified by PCR and cloned into the vector pMGFloxPGUS vector [14] to construct plasmid pΔMGFGUS. PBMs were infected with Benin 97/1 isolate at a multiplicity of infection (MOI) of 3–5 and transfected with plasmid pΔMGFGUS using TRANS-IT LT-1 (Mirus Bio Madison USA). Recombinant viruses expressing the β-GUS gene were identified by incubation of infected cells in the presence of 5-bromo-4-chloro-1H-indol-3-yl β-D-glucopyranosiduronic acid and purified by limiting dilution [13]. The purity of the recombinant virus, BeninΔMGF, was tested by PCR assays (Supplementary Fig. 1). Sequencing of a fragment amplified from across the site of the deletion confirmed the position of the deletion and removal of the first 5 nucleotides of the MGF360-9L gene and the first 7 nucleotides of the MGF530/505-4R genes including the ATG translation start codons and respective promoters (Fig. 1).

Two independently constructed deletion mutants were isolated and confirmed to have the same phenotypes (data not shown).

### 2.3 Analysis of IFN-β transcript levels

PAMs (5 × 10^5^ cells) were infected with ASFV or mock-infected. At selected times, RNA was extracted (RNeasy mini kit, Qiagen, Hilden Germany) with on column DNase I digestion. cDNA was generated using the Superscript III reverse transcriptase kit (Invitrogen, Waltham. MA USA). IFN-β transcripts were measured by quantitative real time PCR (qRTPCR) using power SYBR Green Master Mix (Life Technologies, UK). A DNA blocking oligonucleotide with a ddC modification at the 3′ end was used to avoid amplification of genomic DNA: CCT TCC AGT ATA AAT AGC CTA TGG AGA AAG AAC ATT CAC ACT GCA CA-ddC. The forward primer 5′ terminus overlapped the 3′ terminus of the blocking oligonucleotide (ATT CAC ACT GCA CAC TCC TGA A) and the reverse primer sequence was: GCA CAT CAT AGC TCA TGG AA. PCR amplification included 10 min at 95⁰C, 40 cycles of 30 s at 95 °C, 15 s at 70 °C and 1 min at 65 °C. GAPDH expression was used as internal control, using the primers: TCA ACG ACC ACT TTG TCA AGC and GGT GGT CCA GGG GCT CTT A. IFN-β and GAPDH copy numbers were calculated by the standard curve method and results are presented as the IFN-β/GAPDH ratio. Assays were carried out in duplicate. Transcripts from the ASFV B646L gene were measured by qRTPCR [15].

### 2.4 Immunisation and challenge of pigs

Experiments under Home Office licence PPL 70-6369 were carried out in SAPO4 high containment at The Pirbright Institute using Large White X Landrace pigs (15–20 kg). Clinical signs were monitored daily [7]. Blood samples were collected on different days and tissues (spleen, tonsil, gastro-hepatic, sub-mandibular and mesenteric lymph nodes) were collected at termination.

### 2.5 Quantitative PCR analysis of virus genome copy numbers

DNA was extracted from whole peripheral blood and from tissue samples. Virus genome copy numbers were determined by qPCR [15].

### 2.6 Assay of T cell responses

T cell populations were identified in blood samples using mouse anti-porcine CD4 (clone 74.12.4), CD8 (clone 11.295.33) and gamma delta TCR (clone PPT27) antibodies. Antibodies were incubated with whole blood for 20 min at room temperature and then red blood cells were lysed (FACS lysing solution, BD Biosciences, UK). Cells were pelleted by centrifugation and fixed (4% paraformaldehyde for 30 min). After two washes cells were analysed by flow cytometry (MACSQuant, Miltenyi Biotec. UK). Lymphocyte subsets were gated based on cell surface marker staining using FCS express software (De Novo Software). ASFV specific T cell responses were measured using an IFN-γ ELISPOT assay [7], [16]. Peripheral blood mononuclear cells (PBMC) were stimulated with ASFV or 5 μg/ml phytohaemaglutanin as control.

### 2.7 Measurement of antibody responses against ASFV

Levels of ASFV specific antibodies in serum were measured using a competition ASF ELISA kit (INGENASA PPA3 COMPPAC).

### 2.8 Measurement of cytokine levels in serum

Levels of IL-1β, IL-4, TNFα, IFN-γ and IL-10 in serum were measured using commercially available ELISA kits (R&D Systems, Abingdon, UK).

### 2.9 Statistical analysis

Statistical analysis of temperatures, clinical scores and antibody responses was performed using GraphPad Prism6 software. Significant differences between groups were determined using two-way ANOVA followed by Sidak’s multiple comparison test.

## 3 Results

### 3.1 BeninΔMGF deletion mutant virus replicates with similar kinetics to parental Benin 97/1 and OURT88/3 strains

The contiguous genes MGF360-10L, 11L, 12L, 13L, 14L and MGF530/505-1R, 2R and 3R were deleted and MGF 360-9L and MGF 530/505- 4R interrupted in the genome of the Benin 97/1 virulent isolate (Fig. 1).

**Fig. 2:**
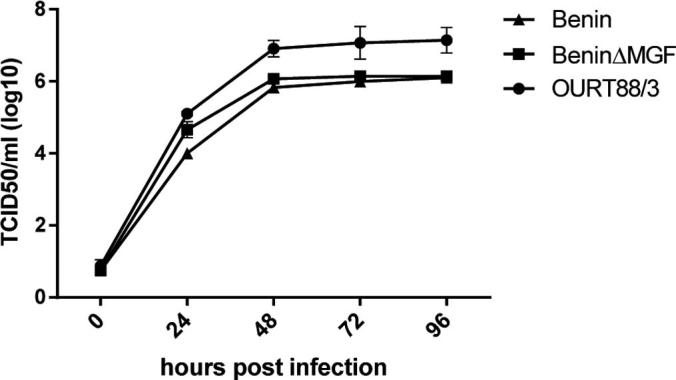
Replication kinetics of Benin 97/1, BeninΔMGF and OURT88/3 viruses. Pig bone marrow macrophages were infected at a low multiplicity of infection (0.3) with Benin 97/1, BeninΔMGF and OURT88/3 isolates as indicated. At various hours post-infection up to 96 h total virus from cells and supernatants was harvested and titrated. Results show means and standard error of the mean (SEM) from two biological replicates using cells from different pigs each of which was assayed in duplicate.

PAMs were infected at a low MOI (0.3) with BeninΔMGF, Benin 97/1 or OURT88/3 strains and total virus harvested at different times was titrated. The results, using macrophages from 2 different pigs, showed similar kinetics of replication reaching a plateau at 48 h post-infection of 1 × 10^6^ for Benin 97/1 and BeninΔMGF and 1 × 10^7^ for OURT88/3 (Fig. 2). Thus deletion of these MGF360 and MGF530/505 genes from the Benin 97/1 genome did not significantly alter the virus replication kinetics in macrophages.

### 3.2 Levels of IFN-β mRNA in porcine macrophages are increased following infection with OURT88/3 and BeninΔMGF

**Fig. 3:**
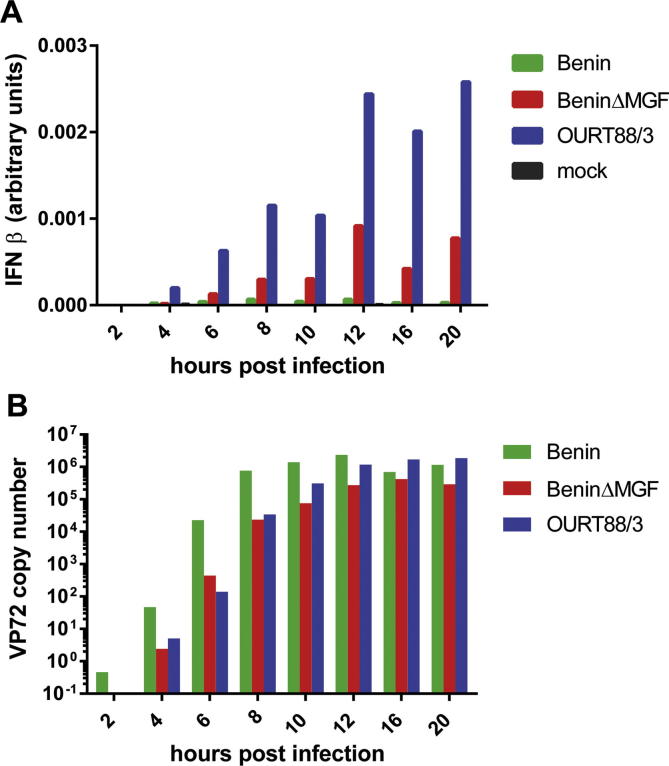
Amounts of mRNA for IFN-β and ASFV VP72 (B646L) gene detected at different times post-infection of macrophages with Benin 97/1, BeninΔMGF or OURT88/3 strains. Porcine alveolar macrophages were infected at a MOI of 1 or mock-infected. At different times post-infection (*x*-axis) RNA was extracted and Panel A shows the amounts of IFN-β mRNA determined by qRTPCR in comparison to GAPDH (*y*-axis). Panel B shows copy numbers of VP72 mRNA (B646L gene) estimated in comparison to control plasmid analysed in parallel. The graph shows typical results obtained from one of three biological replicates using cells from a single pig. Each sample was assayed in duplicate.

PAMs were infected at a MOI of 1 with Benin 97/1, BeninΔMGF, OURT88/3 virus strains or were mock-infected. Levels of mRNA for porcine IFN-β and the ASFV B646L late gene were determined by qRTPCR at different times post-infection. Fig. 3 shows typical results from one of 3 biological replicates using macrophages from different outbred pigs. Levels of mRNA for the VP72 B646L gene were similar in infections with the different viruses confirming that similar levels of infection were obtained. These reached a plateau of 10^5^–10^6^ copy numbers by 10–12 h post-infection (Fig. 3B).

IFN-β mRNA was detected at very low levels or was not detected in mock-infected cells and in cells infected with Benin 97/1 (Fig. 3A). In contrast high levels of IFN-β mRNA were detected in macrophages infected with OURT88/3. Intermediate levels were detected in macrophages infected with BeninΔMGF, varying in different biological replicates between 6 and 20% of that detected at 16 h following OURT88/3 infections. Low levels of IFN-β mRNA were detected from 4 h post-infection reaching a peak by 12 h which was maintained during the remainder of the time course. Thus deletion of the members of MGF360 and 530/505 resulted in an increase in IFN-β gene transcripts, indicating that these genes have a role in suppressing IFN-β induction.

### 3.3 The BeninΔMGF deletion mutant virus is attenuated in pigs and induces a protective response against challenge with parental virus: Clinical and post-mortem signs

**Fig. 4:**
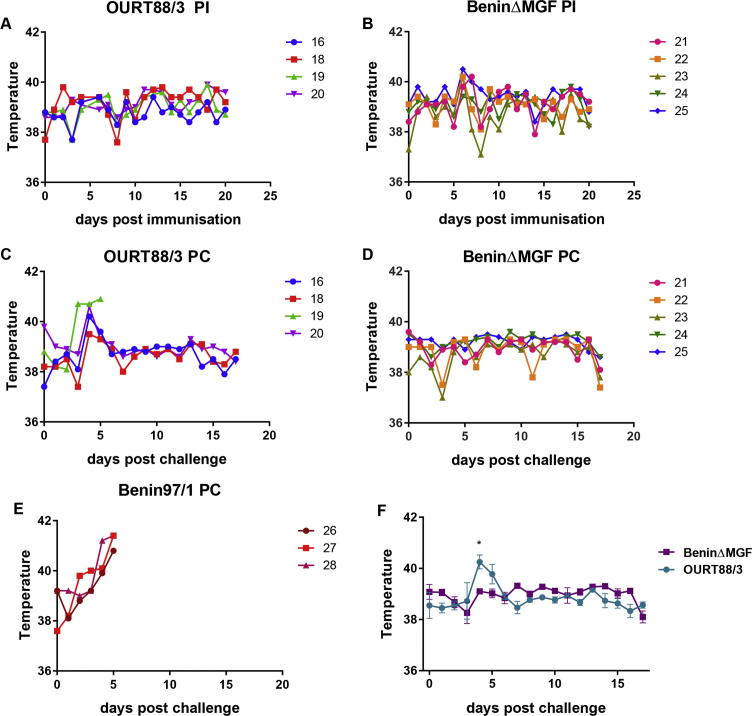
Temperatures after inoculation and challenge of pigs. Panel A and B show temperatures (*y*-axis) of individual pigs at different days post-inoculation (PI) (*x*-axis) with OURT88/3 or BeninΔMGF respectively. Panels C and D show temperatures of individual pigs (*y*-axis) at different days post-challenge (PC) (*x*-axis) with OURT88/3 or BeninΔMGF respectively. Panel E shows temperatures (*y*-axis) of control non-immune pigs at different days post-challenge with Benin 97/1 (PC) (*x*-axis). Panel F shows means and standard error of the mean (SEM) of temperatures (*y*-axis) of pigs inoculated with OURT88/3 (teal) or BeninΔMGF (purple) at different days post-challenge (*x*-axis). Asterisk indicates results significantly different (^*^P ⩽ 0.05) between groups.

Five pigs were inoculated with the BeninΔMGF virus (10^2^ TCID_50_) and four pigs with the attenuated OURT88/3 isolate (10^4^ TCID_50_) by the intramuscular (IM) route. After 25 days the pigs were boosted with the same viruses IM using 10^4^ TCID_50_. At day 46, the pigs were challenged with the parental Benin 97/1 virus (10^4^ TCID_50_ IM) in parallel with three control non-immune pigs. Four pigs inoculated with BeninΔMGF developed a transient fever of 40–40.5 °C for 1 day (21, 22, 23) or 2 days (25) at day 6 or 7 post-inoculation (Fig. 4). No clinical signs were observed after challenge with virulent virus.

**Supplementary Fig. 2:**
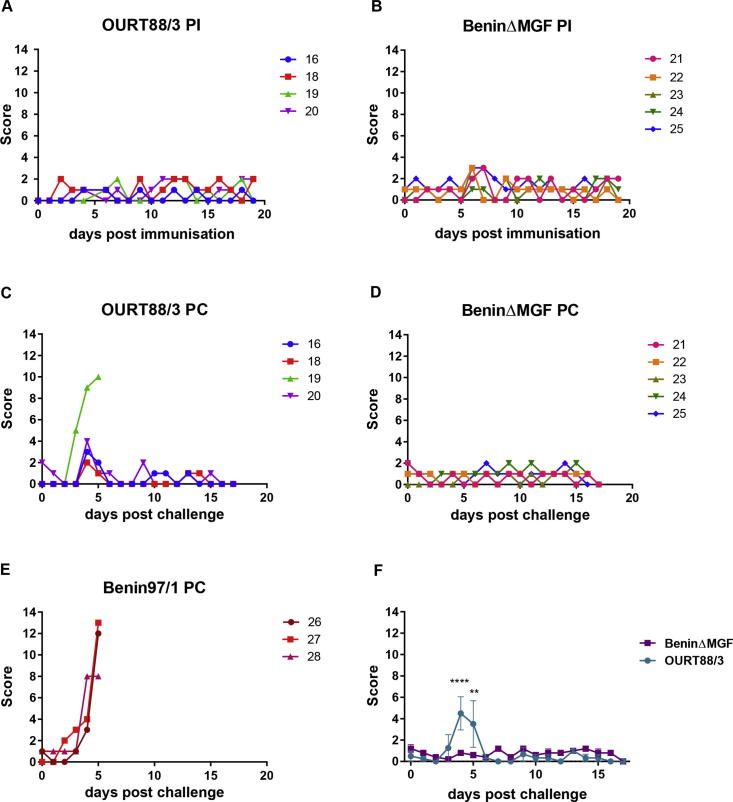


No clinical signs were observed in pigs post-immunisation with OURT88/3. Following challenge with virulent Benin 97/1 virus, one of the pigs (19) developed fever and other clinical signs characteristic of acute ASF (lethargy, inappetence) at day 3 post-challenge and was terminated at day 5 post-challenge. Two of the other pigs (16, 20) in this group had a transient fever of 40–40.5 °C at day 4 post-challenge. In summary, pigs immunised with OURT88/3 showed significantly higher temperatures (p = 0.0118) (Fig. 4F) and clinical scores (Supplementary Fig. 2F) than pigs immunised with BeninΔMGF after challenge. The control group of non-immune pigs all developed clinical signs typical of acute ASF and were terminated at day 5 post-challenge. All remaining pigs were terminated at day 17 post-challenge. Post-mortem examination of pigs showed lesions typical of acute ASFV in the three non-immune control pigs (26, 27, 28) and in pig 19. These included an enlarged haemorrhagic spleen and lymph nodes.

### 3.4 Virus load in blood and tissues

**Fig. 5:**
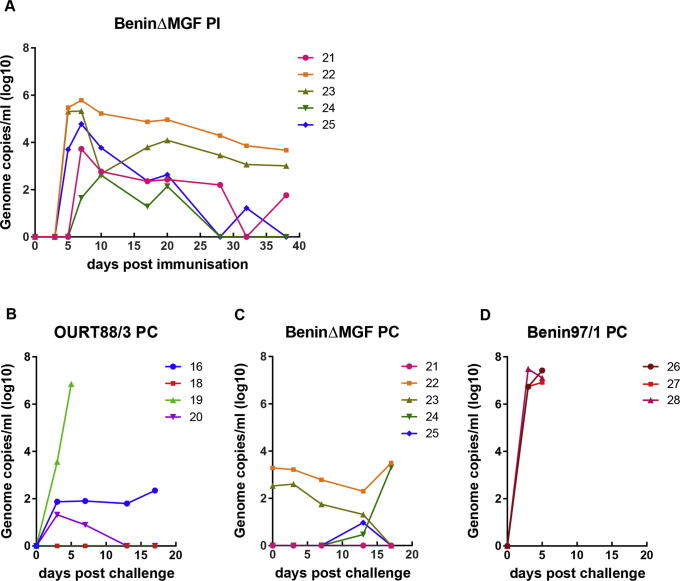
Virus genome copies detected in blood and tissues. Blood samples were collected at different days post-inoculation (PI) or post-challenge (PC) with Benin 97/1 (*x*-axis) and amounts of virus DNA present determined by qPCR in comparison with a control plasmid (*y*-axis). Panel A shows virus genome copies per ml in blood from individual pigs post-inoculation with BeninΔMGF. No virus DNA was detected in blood from pigs post-inoculation with OURT/88/3 strain. Panels B, C, D show virus genome copies per ml of blood detected in individual pigs inoculated with OURT88/3 (Panel B), BeninΔMGF (Panel C) and non-immune pigs (Panel D) post-challenge with Benin 97/1. Results shown are averages of duplicate samples.

Virus genome (10^2^–10^6^ copies per ml) was detected in blood from all of the pigs’ post-immunisation with the BeninΔMGF virus. This was detected from day 5 or 7 post-immunisation until days 28–46 and declined over this period to between 10^4^ genome copies per ml and undetectable levels. Low levels of virus genome (<10^4^ genome copies per ml) were detected in four of the pigs post-challenge (Fig. 5). None of the pigs immunised with OURT88/3 had detectable levels of virus DNA in blood post-immunisation (data not shown). Virus genome was detected (10^6^ genome copies per ml) in blood at day 5 post-challenge from the one pig (19) that was not protected following challenge. As expected the 3 non-immune pigs (26, 27, 28) had high levels of ASFV genome in blood from the onset of clinical signs at day 3 post-challenge (10^6^–10^7^ genome copies per ml) and were terminated at day 5 post-challenge (Fig. 5D).

Virus genome was detected in the spleen of pig 24 (2.4 × 10^3^ copies per g tissue) and in the sub-mandibular lymph node of pig 25 (7.8 × 10^2^ copies per g tissue) but not in the other tissues collected or in samples from other pigs. Virus genome levels typical for acute ASF (between 10^6^ and 10^8^ genome copies per g of tissue) were detected in all 5 tissues analysed from the control non-immune pigs (data not shown).

### 3.5 T cell responses in immunised pigs: IFN-γ ELIspot assays

**Supplementary Fig. 3:**
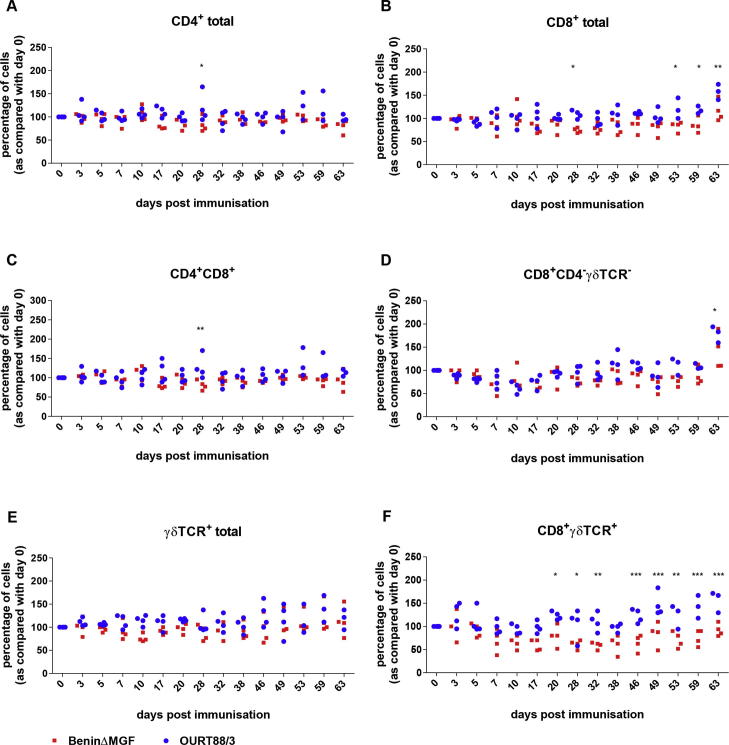


**Fig. 6:**
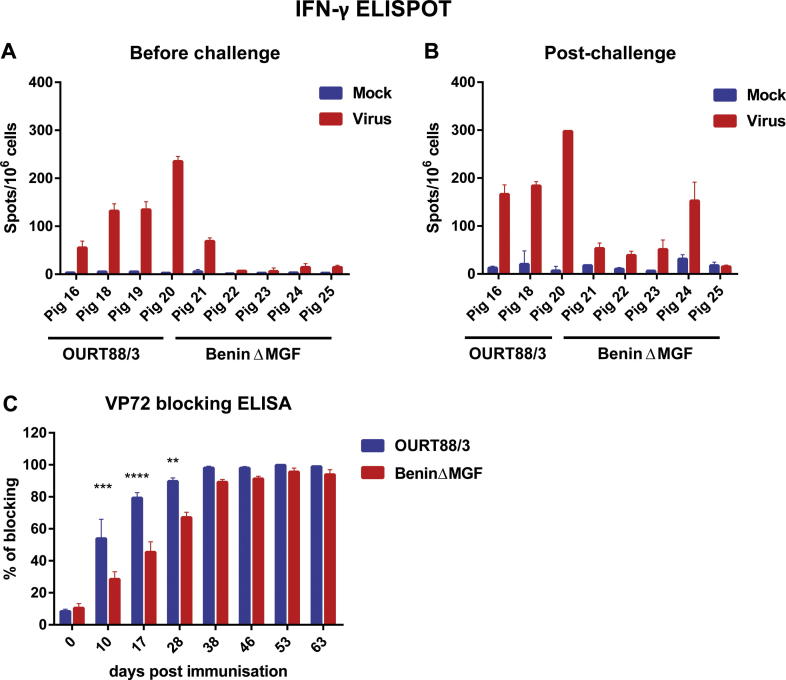
Immune responses in vaccinated pigs. Panels A and B show numbers of IFN-γ producing cells by ELISPOT assays using PBMC stimulated with ASFV Benin 97/1. PBMC isolated on day 46 post-inoculation (panel A) or day 17 post-challenge (panel B) with OURT88/3 or BeninΔMGF were stimulated *ex vivo* with either medium alone or Benin 97/1 virus. Results show mean and SD of numbers of IFN-γ producing cells per 10^6^ lymphocytes (*y*-axis) and type of stimulation (*x*-axis) for the different pigs. Panel C shows the antibody response to ASFV post-immunisation and challenge. Sera were collected at different days post-inoculation (*x*-axis) of pigs with OURT88/3 or BeninΔMGF strains. Levels of anti-VP72 antibodies were detected using a blocking ELISA assay. The percentage of blocking is shown (*y*-axis). Results show mean and SEM for the different groups of pigs and asterisks indicate days on which statistically significant differences were observed between the groups of pigs (^**^P ⩽ 0.01; ^***^P ⩽ 0.001; ^****^P ⩽ 0.0001).

The responses of lymphocytes from immunised pigs were measured following stimulation of PBMC with live Benin 97/1 isolate or medium (Fig. 6A and B). High numbers of IFN-γ producing cells were detected following virus stimulation of PBMC isolated before challenge and at 17 days post-challenge from pigs immunised with OURT88/3. Responses to medium alone were very low. In contrast generally low numbers of IFN-γ producing cells were induced by virus stimulation of PBMC from pigs which were immunised with BeninΔMGF. Before challenge only one pig (no 21) showed numbers of IFN-γ producing cells similar to those observed from pigs immunised with OURT88/3. These results indicated there may be a lower specific T cell response to ASFV in pigs immunised with BeninΔMGF compared to OURT88/3. To further investigate we measured changes in the proportion of different subpopulations of lymphocytes over the course of the experiment (results shown as a percentage of the numbers observed at day 0) (Supplementary Fig. 3). Pigs infected with BeninΔMGF showed significantly lower numbers of circulating CD8^+^ gamma delta T cells as early as day 20 post-infection. No other major differences between OURT88/3 and BeninΔMGF infected animals were observed for the other cell populations studied.

### 3.6 Antibody responses in immunised pigs

Anti-ASFV VP72 antibodies (Fig. 6C) were first detected from day 10 post-immunisation and increased until day 38 remaining at the same level until the experiment was terminated at day 63. Levels of anti-VP72 antibodies were significantly lower in pigs immunised with BeninΔMGF compared to OURT88/3 on days 10 (p = 0.0006), 17 (p < 0.0001) and 28 (p = 0.0026) post-immunisation. No virus neutralisation was detected using serum collected on day 63 from any of the immunised pigs (data not shown).

### 3.7 Cytokine levels in serum

**Supplementary Fig. 4:**
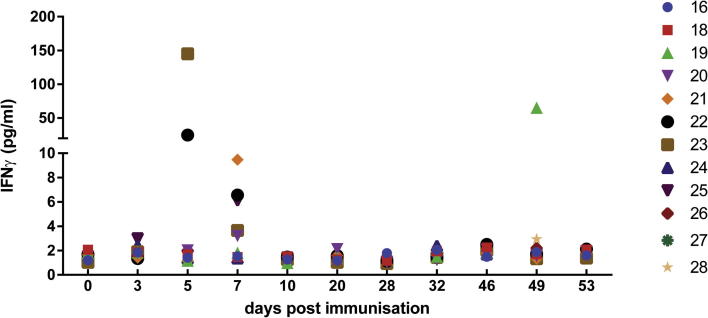


Comparison of the levels of cytokines in sera from pigs immunised with BeninΔMGF and OURT88/3 showed an increase in the amount of IFN-γ detected at days 5 and 7 post-immunisation in four of the pigs immunised with BeninΔMGF (Supplementary Fig. 4). This increase was not observed in pigs infected with the OURT88/3 isolate. At other days tested IFN-γ levels remained low, with the exception of the pig that was infected with OURT88/3 and did not survive challenge. In this pig there was a significant increase in IFN-γ three days post-challenge. There were no significant differences observed in levels of IL-1β, IL-4, TNFα or IL-10 in pigs infected with BeninΔMGF compared to OURT88/3 (data not shown).

## 4 Discussion

We extended studies on the impact of deleting or interrupting multiple copies of genes belonging to MGF360 and MGF530/505 on virus replication and type I IFN induction in macrophages in culture and virus pathogenesis and induction of protection in pigs. The genes deleted or interrupted from the virulent Benin 97/1 isolate (MGF360-9L, 10L, 11L, 12L, 13L, 14L MGF530/505 -1R, 2R, 3R, 4R) included one gene (MGF530/505-4R) in addition to those deleted from the attenuated OURT88/3 isolate [9] and 3 in addition to those from Pr4 and Georgia [10], [11]. Our results indicate that these genes are involved in suppressing type I IFN induction in infected macrophages in culture since IFN-β mRNA was consistently observed in macrophages infected with the deletion mutant but not those infected with the virulent parental virus Benin 97/1. Higher levels of IFN-β mRNA were consistently induced in macrophages infected with OURT88/3 compared to BeninΔMGF suggesting that other genes altered in the OURT88/3 isolate have a similar role. These may include MGF360-5L and MGF530/505-5R, since these are present in Benin 97/1 and BeninΔMGF but not in OURT88/3 [9]. The mechanism by which the MGF360 and MGF530/505 genes act is unknown. The relatively high sequence divergence between different gene copies suggests they could have different molecular targets.

The numbers of MGF360 and 530/505 genes encoded by different isolates vary and thus the same gene deletions may vary in effect dependent on the isolate. Our results confirm that the deletion of this subset of MGF360 and 530/505 genes from a virulent genotype I isolate results in virus attenuation and induction of protection. Thus this deletion may be used to rationally attenuate virulent isolates from any ASFV genotype. We demonstrated a correlation between increased induction of type I IFN-β mRNA in macrophages infected *in vitro* and virus attenuation in pigs. Replication of virulent isolates in macrophages is resistant to pre-treatment with high levels of IFN-α whereas OURT88/3 isolate was slightly sensitive (∼10^2^ reduction in titre) [17]. Thus the induced type I IFN may contribute to control replication of OURT88/3 and BeninΔMGF by inducing an antiviral response and by activating innate immunity and thus reduce virus pathogenesis in pigs. The higher levels of virus genome detected in blood following immunisation of pigs with BeninΔMGF compared to OURT88/3 may result from the intact EP402R gene in BeninΔMGF. This gene encodes the CD2v protein which is required for binding of virus particles and infected cells to red blood cells. This is predicted to result in binding of extracellular virions to red blood cells and thus increase levels of virus detected in blood. This may also result in increased dissemination of virus in pigs [18], [19]. Although we did not detect virus genome in blood post-immunisation with OURT88/3 transient viremia may have been missed in intervals between sampling. Other genome differences have been described between OURT88/3 and Benin 97/1 [9] and may also contribute to differences between the clinical signs and protection induced by the two viruses.

In an attempt to identify correlates of protection the immune responses were compared between pigs immunised with BeninΔMGF and OURT88/3, a well-characterised model for induction of protection. As previously observed [7], immunisation with OURT88/3 results in the induction of high numbers of IFN-γ producing lymphocytes following stimulation with ASFV. Interestingly, pig 19 although showing similar responses to the other pigs inoculated with OURT88/3 did not survive challenge. The lower numbers of IFN-γ producing lymphocytes observed following stimulation of lymphocytes from the pigs immunised with BeninΔMGF were not explained by a general depletion in numbers of lymphocytes. No significant differences in the proportions of total CD4^+^, CD8^+^, CD4^+^CD8^+^ and total gamma delta T cells were observed between the OURT88/3 and BeninΔMGF immunised pigs. Alternatively, the prolonged viremia observed in pigs immunised with BeninΔMGF could be associated with the decreased IFN-γ T cell responses. However results obtained in our laboratory using another deletion mutant in the same Benin 97/1 background, showed high numbers of IFN-γ producing cells in pigs with similar high viremias (Reis et al., unpublished data). Taken together, these results suggest that in addition to IFN-γ production, protection may be correlated with induction of a different T cell response.

Pigs immunised with BeninΔMGF showed an increase in serum IFN-γ at days 5 and 7 post infection. This cytokine was most probably secreted by NK or NKT cells, since it was too early for the development of specific T lymphocytes. IFN-γ plays an import role in the establishment of the adaptive immunity, for example by enhancing MHC class II presentation and favouring a Th1 type response, and therefore may have had a positive impact in protection.

Our results indicate that deletion of IFN modulators is a promising route for rational attenuation of virulent ASFV to produce candidate vaccine strains.

## Conflict of interest

The authors declare they have no conflict of interest.
